# Unrelated donor hematopoietic stem cell transplantation compared to immunosuppressive therapy plus eltrombopag as first-line treatment for adults with severe aplastic anemia

**DOI:** 10.1038/s41408-024-01021-x

**Published:** 2024-03-06

**Authors:** Liangliang Wu, Limin Liu, Xin Zhao, Ming Zhou, Andie Fu, Yuping Zhang, Wenrui Yang, Xiaowei Chen, Wenjian Mo, Caixia Wang, Yumiao Li, Shilin Xu, Shiyi Pan, Ruiqing Zhou, Fankai Meng, Fengkui Zhang, Depei Wu, Shunqing Wang

**Affiliations:** 1 Department of Hematology, Guangzhou First People’s Hospital, South China University of Technology, Guangzhou, Guangdong China; 2 National Clinical Research Center for Hematologic Diseases, Jiangsu Institute of Hematology, The First Affiliated Hospital of Soochow University, Institute of Blood and Marrow Transplantation of Soochow University, Suzhou, Jiangsu China; 3 State Key Laboratory of Experimental Hematology, National Clinical Research Center for Blood Diseases, Anemia Therapeutic Center, Institute of Hematology & Blood Diseases Hospital, Chinese Academy of Medical Sciences & Peking Union Medical College, Tianjin, China; 4 Department of Hematology, Tongji Hospital, Tongji Medical College, Huazhong University of Science and Technology, Wuhan, Hubei China

**Keywords:** Anaemia, Quality of life

Allogeneic hematopoietic stem cell transplantation (allo-HSCT) and immunosuppressive therapy (IST) are the main therapeutic options for severe aplastic anemia (SAA) [1]. The first-line therapy for < 35-year-old adults with SAA is HLA-matched sibling donor (MSD) HSCT [1]. IST is the traditionally recommended first-line therapy for adult patients not eligible for MSD-HSCT [1]. Recently, eltrombopag (EPAG) plus IST as a front-line treatment improved the rate and rapidity of the hematologic response [2]. However, patients treated with IST + EPAG are at risk of severe infection, bleeding and clonal hematopoiesis [3]. Unrelated donor HSCT (URD-HSCT) for adults with SAA is currently recommended after IST failure [1]. Encouragingly, as transplantation technology advances, the survival outcomes of URD-HSCT have improved dramatically [4, 5]. The above has stimulated the transplantation community to consider giving URD-HSCT a larger role as a first-line option for adult SAA patients who lack an MSD [4–8]. However, no studies have compared the outcomes of first-line URD-HSCT and IST + EPAG in adults.

Aside from survival, hematologic recovery and health-related quality of life (HRQoL) are major concerns for survivors [9, 10]. However, there has been no research comparing these metrics between first-line UDR-HSCT and IST + EPAG. We conducted a multicenter retrospective cohort study to compare the outcomes of adults with SAA who underwent URD-HSCT or IST + EPAG as an upfront treatment to determine whether upfront treatment with URD-HSCT or IST + EPAG is a better option for adults with SAA who lack an MSD by focusing on survival outcomes, hematologic response and HRQoL.

One hundred fourteen patients who received upfront URD-HSCT and 99 patients who received initial IST + EPAG treatment from November 2012 to October 2022 were enrolled. The details of patient selection, URD-HSCT and IST + EPAG protocols, evaluation of HRQoL, definition of hematologic response and survival outcome, and statistical analyses are in the Supplementary methods. The date of the last follow-up for survivors was April 30, 2023. The study received local review board approval. Written informed consent was obtained from the patients or their caretakers under the Declaration of Helsinki.

Patients treated with URD-HSCT were younger than patients treated with IST + EPAG (*p* = 0.023). The treatment times had different distributions (*p* < 0.001). There was no difference in the male/female ratio or disease severity. Additional characteristics of patients are summarized in Table 1.

**Table 1:** Comparison of patient characteristics in the IST + EPAG and URD-HSCT groups.

Characteristic	Before PSM		*P*	After PSM		*P*
	IST + EPAG	URD-HSCT		IST + EPAG	URD-HSCT	
	(*N* = 99)	(*N* = 114)		(*N* = 66)	(*N* = 66)	
Age at treatment, *y*, median (range)	31.0 (15–55)	27.5 (15–54)	0.023	29.0 (16–55)	28.5 (15.0–54.0)	0.158
Age, *y*, *n* (%)			0.015			0.518
15–20	23 (23.2)	28 (24.6)		13 (19.7)	16 (24.2)	
21–35	40 (40.4)	64 (56.1)		29 (43.9)	32 (48.5)	
36–55	36 (36.4)	22 (19.3)		24 (36.4)	18 (27.3)	
Sex, *n* (%)			0.823			1
Female	45 (45.5)	49 (43.0)		32 (48.5)	31 (47.0)	
Male	54 (54.5)	65 (57.0)		34 (51.5)	35 (53.0)	
Diagnosis, *n* (%)			0.197			0.353
NSAA	0 (0)	4 (3.5)		0 (0)	2 (3.0)	
SAA	63 (63.6)	68 (59.6)		41 (62.1)	41 (62.1)	
VSAA	36 (36.4)	42 (36.8)		25 (37.9)	23 (34.8)	
Interval from diagnosis to treatment, m, median (range)	1.0 (0.2–6.0)	4.0 (0.5–6.0)	<0.001	1.0 (0.2–6.0)	4.0 (1.0–6.0)	<0.001
Period of treatment, *n* (%)			<0.001			0.718
11/2012–12/2017	3 (3.0)	42 (36.8)		3 (4.5)	5 (7.6)	
1/2018–10/2022	96 (97.0)	72 (63.2)		63 (95.5)	61 (92.4)	
Follow-up time among alive patients, d, median (range)	970 (257–2103)	1467 (194–3824)	<0.001	990 (257–2103)	1028 (194–2440)	0.834
OR at 3-month, *n* (%)	50 (50.5)	109 (95.6)	<0.001	34 (51.5)	64 (97.0)	<0.001
OR at 6-month, *n* (%)	67 (67.7)	109 (95.6)	<0.001	45 (68.2)	65 (98.5)	<0.001
Relapse, *n* (%)	2 (1.7)	2 (1.8)	0.887	1 (1.5)	0 (0)	0.316
Median neutrophil count to reach ≥ 1 × 10^9^/L, days (range)	52.0 (6–148)	13.0 (9–29)	<0.001	58.5 (6–148)	13.0 (9–20)	<0.001
HLA typing, *n* (%)	-	-	-	-	-	-
10/10	-	86 (75.4)	-	-	53 (80.3)	-
9/10	-	28 (24.6)	-	-	13 (19.7)	-
HLA mismathing locus, *n* (%)	-		-	-		-
HLA-A	-	9 (7.9)	-	-	4 (6.1)	-
HLA-B	-	2 (1.8)	-	-	1 (1.5)	-
HLA-C	-	10 (8.8)	-	-	3 (4.5)	-
HLA-DQB1	-	4 (3.5)	-	-	2 (3.0)	-
HLA-DRB1	-	3 (2.6)	-	-	3 (4.5)	-
Conditon regimen, *n* (%)	-		-	-		-
BUCy	-	47 (41.2)	-	-	41 (62.1)	-
FCA	-	46 (40.4)	-	-	25 (37.9)	-
PTCy-TBI		14 (12.3)	-		-	-
PTCy-Bu	-	7 (6.1)	-	-	-	-
ABO match, n(%)	-		-	-		-
Matched	-	44 (38.6)	-	-	29 (43.9)	-
Minor mismatched	-	28 (24.6)	-	-	18 (27.3)	-
Major mismatched	-	30 (26.3)	-	-	14 (21.2)	-
Different	-	12 (10.5)	-	-	5 (7.6)	-
MNC, 10^8^/kg, median (range)	-	9.2 (3.5–19.2)^a^	-	-	9.41 (4.30–19.2)	-
CD34^+^ cell count, 10^6^/kg, median (range)	-	5.0 (0.26–19.8)^b^	-	-	5.08 (0.26–9.80)	-
Donor age, y, median (range)	-	30 (20–47)^c^	-	-	28.0 (20–46)^d^	-
Donor-Recipient sex, *n* (%)	-		-	-		-
Female–female	-	4 (3.5)	-	-	1 (1.5)	-
Female–male	-	13 (11.4)	-	-	6 (9.1)	-
Male-female	-	43 (37.7)	-	-	29 (43.9)	-
Male–male	-	54 (47.4)	-	-	30 (45.5)	-
Neutrophil engraftment time, *d*, median (range)	-	11 (8–24)	-	-	11 (9–16)	-
Platelet engraftment time, *d*, median (range)	-	12 (6–98)	-	-	12 (6–98)	-
PGF, *n* (%)	-	2 (1.8)	-	-	1 (1.5)	-
SGF, *n* (%)	-	2 (1.8)	-	-	0	-

*NSAA* non-severe aplastic anemia; *PGF* primary graft failure, *SGF* sencondary graft failure.

^a^Two patient’s data unknown.

^b^One patient’s data unknown.

^c^Six donor’s age unknown.

^d^Three donor’s age unknown.

Of the 114 patients in the URD-HSCT cohort, 112 achieved neutrophil and platelet engraftment. The median time to neutrophil and platelet engraftment was 11.0 days (range, 8–24) and 12.0 days (range, 6–98), respectively. The cumulative incidence (CuI) of both neutrophil and platelet engraftment was 98.2 ± 1.4% (Supplementary Fig. 1). The CuI of graft failure was 3.5 ± 1.7%. The CuI of grade II-III aGVHD and cGVHD were 16.1 ± 3.5% and 11.8 ± 3.1%, respectively. The CuI of grade II-III aGVHD was significantly higher in the MMUD-HSCT (HLA 9/10 URD) group than in the MUD-HSCT (HLA 10/10 URD) group (*p* = 0.010). There was no significant difference in the CuI of cGVHD between the MUD and MMUD groups (*p* = 0.912) (Supplementary Fig. 1). Thirteen patients died after transplantation. There was a median follow-up of 1467 days (range, 194–3824) among the surviving patients, with both overall survival (OS) and failure-free survival (FFS) rates of 86.5% (95% CI 79.7–93.8). GVHD-free, failure-free survival (GFFS) was 83.2% (95% CI 76.0–91.0). Moreover, the hematologic status of patients surviving with GFFS was a complete response (CR). No significant difference was found in OS or GFFS between the MUD and MMUD groups (Supplementary Fig. 2).

In the IST + EPAG group, patients received EPAG for a median of 10 months (range, 1.5–22.2) at a median dosage of 100 mg/day (range, 50–150). The overall response (OR) rate was 50.5% (50/99) at 3 months, including 4.0% (4/99) with CR and 46.5% (46/99) with partial response (PR). The OR was 67.7% (67/99) at 6 months, including 22.2% (22/99) with CR and 45.5% (45/99) with PR. Nine patients underwent salvage HSCT, one of whom died after transplantation. Seven patients died after treatment with IST + EPAG. The OS and FFS at 5 years were 92.4% (95% CI, 87.2–98.0) and 67.7% (95% CI 59.1–77.5), respectively. Survival with CR status (CROS) at 4 years was 29.3% (95% CI 21.6–39.8).

The OR was higher after URD-HSCT than IST + EPAG at 3 months (*p* < 0.001) and 6 months (*p* < 0.001) (Table 1). Patients who underwent URD-HSCT achieved an absolute neutrophil count ≥1.0×10^9^/L faster than those who underwent IST + EPAG (*p* < 0.001) (Table 1). No significant difference was found in OS at five years between groups (*p* = 0.362). In the subgroup analysis, among patients aged 15–20 years, 21–35 years, or 36–55 years and for patients with SAA or very SAA (VSAA), OS at 5 years was also not significantly different between groups (Supplementary Fig. 3). FFS at 5 years after URD-HSCT was markedly superior to that after IST + EPAG (*p* < 0.001) (Fig. 1A). Subgroup analysis of patients aged 15–20 years revealed no significant difference in FFS at 5 years (*p* = 0.682) (Fig. 1B). Among patients aged 21–35 years (*p* = 0.007), 36–55 years (*p* = 0.009), SAA (*p* = 0.001) and VSAA (*p* = 0.026), FFS at five years was significantly better after URD-HSCT than after IST-EPAG (Fig. 1C–F). Moreover, the GFFS at 4 years after URD-HSCT was also significantly higher than that of CROS at 4 years after IST + EPAG (*p* < 0.001) (Fig. 1G). Subgroup analysis of patients aged 15–20 years (*p* < 0.001), 21–35 years (*p* < 0.001), or 36–55 years (*p* < 0.001), with SAA (*p* < 0.001), or with VSAA (*p* < 0.001), GFFS after URD-HSCT was significantly better than the CROS after IST-EPAG (Fig. 1H-L). The HRs from the subgroup and univariable analyses are listed in Supplementary Tables 1 and 2. The multivariable analyses showed that choosing upfront URD-HSCT was a favorable factor for FFS (HR 0.204, 95% CI 0.077–0.542, *p* = 0.001) and for GFFS/CROS (HR 0.073, 95% CI 0.033–0.163, *p* < 0.001) but did not affect OS (Supplementary Table 2).

**Fig. 1:**
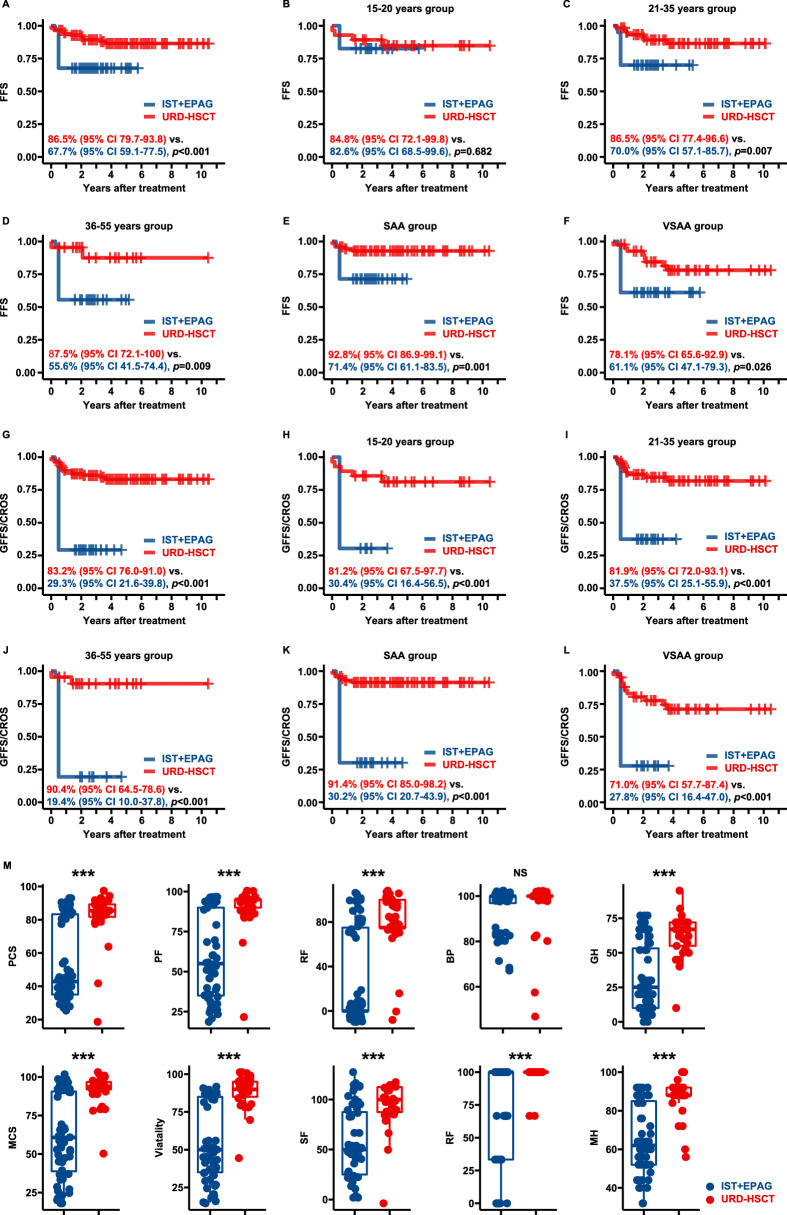
Survival outcomes and health-related quality of life in SAA patients who underwent URD-HSCT or IST + EPAG. **A** FFS in the two cohorts; **B** FFS in 15–20 years group; **C** FFS in 21–35 years group; **D** FFS in 36–55 years group; **E** FFS in SAA group; **F** FFS in VSAA group; **G** GFFS/CROS in the two cohorts; **H** GFFS/CROS in the 15–20 years group; **I** GFFS/CROS in the 21–35 years group; **J** GFFS/CROS in the 36–55 years group; **K** GFFS/CROS in SAA group; **L** GFFS/CROS in VSAA group; **M** health-related quality of life in the two groups. PCS physical component summary, PF physical functioning, RF role-physical functioning, BP bodily pain, GH general health, MCS mental component summary, SF social functioning, RF role-emotional functioning, MH mental health.

Thirty-three URD-HSCT patients and 56 IST + EPAG patients alive at the end of follow-up participated in the HRQoL study. Except for bodily pain, patients who underwent URD-HSCT reported significantly higher physical component summary, physical functioning, role-physical functioning, general health, mental component summary, vitality, social functioning, role-emotional functioning and mental health scores (Fig. 1M, Supplementary Table 3). In the multiple linear regression, URD-HSCT as treatment was the only favorable factor for HRQoL (Supplementary Table 4). Moreover, the patients who achieved CR had a significantly higher score than patients who achieved PR in two groups (Supplementary Table 5).

To minimize confounding factors between two groups, propensity score matching (PSM) was applied based on 2 variables (patient age at treatment and period of treatment) [11]. All variables except the time interval between diagnosis and treatment, which can be explained by more time needed to find a donor, were balanced between the 2 cohorts after PSM (Table 1). Consistent with the results before PSM, no significant difference was found in OS after PSM (Supplementary Fig. 3). After PSM, FFS of patients who underwent URD-HSCT was also superior to that of patients who underwent IST + EPAG overall (*p* < 0.001) and at 21–35 years (*p* = 0.002) and SAA subgroup (*p* < 0.001). Excellent FFS in the URD-HSCT group was also found at 15–20 years (*p* = 0.071), 36–55 years (*p* = 0.081) and VSAA (*p* = 0.104), although these differences were not statistically significant (Supplementary Fig. 4). GFFS/CROS in the URD-HSCT group was still superior to that in the IST + EPAG group overall and in the subgroup analysis (Supplementary Fig. 4). In the multivariate analysis, the choice of upfront URD-HSCT was still a favorable factor for FFS and GFFS/CROS (Supplementary Table 2). In line with the findings before PSM, except for bodily pain, patients who underwent URD-HSCT reported significantly higher scores for other components (Supplementary Fig. 5, Supplementary Table 3). URD-HSCT was still a favorable factor for HRQoL according to multiple linear regression (Supplementary Table 4). After PSM, the score of patients who achieved a CR was still higher than that of patients who achieved a PR (Supplementary Table 5).

As in previous study of MSD-HSCT versus IST + EPAG [12], the hematologic response rate and speed of URD-HSCT were higher and faster than those of IST + EPAG, and URD-HSCT and IST + EPAG yielded similar OS rates in the present study. However, the improved OS in the first-line IST + EPAG group may be partly derived from the fact that patients did not respond to initial IST + EPAG and subsequently underwent salvage transplantation [12]. Even so, our data still indicate that FFS and HRQoL in the URD-HSCT group are superior to those in the IST + EPAG group. As shown previously [13, 14], cGVHD has a significant adverse effect on HRQoL in transplant recipients. In this study, only 11.8% of patients experienced cGVHD. Moreover, our data indicate that the hematologic response also correlates with HRQoL. The CROS after IST + EPAG was significantly lower than the GFFS after URD-HSCT. The impaired HRQoL in the IST + EPAG group was attributed, at least in part, to not achieving a CR.

In summary, our data indicate that in adults with SAA without an MSD, upfront URD-HSCT yields better FFS, GFFS/CROS and HRQoL than IST + EPAG. The role of URD-HSCT in the SAA treatment algorithm may be considered for adult patients who lack an MSD. Due to the retrospective nature of our study and the inferiority of rabbit antithymocyte globulin (ATG) to horse ATG [15], prospective research comparing upfront URD-HSCT to the triple combination of horse ATG, cyclosporine and eltrombopag is needed.

### Supplementary information

Supplementary Material

## Data Availability

The data that support the findings of this study are available from the corresponding author upon reasonable request.
