# Supplementary material for: Unrelated donor hematopoietic stem cell transplantation compared to immunosuppressive therapy plus eltrombopag as first-line treatment for adults with severe aplastic anemia
Source: Blood Cancer J. 2024 Mar 6;14(1):37. doi: 10.1038/s41408-024-01021-x (PMC10914753; doi:10.1038/s41408-024-01021-x)
Supplement: Supplementary file 1 — Supplementary Material [file 41408_2024_1021_MOESM1_ESM.pdf]

Unrelated donor hematopoietic stem cell transplantation compared to immunosuppressive therapy plus eltrombopag as first-line treatment for adults with severe aplastic anemia

Liangliang Wu,[1,5] Limin Liu,[2,5] Xin Zhao,[3,5] Ming Zhou,[1,5] Andie Fu,[4,5] Yuping Zhang,[1] Wenrui Yang,[3] Xiaowei Chen,[1] Wenjian Mo,[1] Caixia Wang,[1] Yumiao Li,[1] Shilin Xu,[1] Shiyi Pan,[1] Ruiqing Zhou,[1] Fankai Meng,[4,6] Fengkui Zhang,[3,6] Depei Wu,[2,6] and Shunqing Wang,[1,6]

Methods and Patients

URD-HSCT and IST+EPAG cohorts

All patients in this study met the following eligibility criteria: (1) AA acquired according to the criteria; [1] (2) interval from diagnosis to treatment (URD-HSCT or IST+EPAG) ≤6 months; (3) age > 14 years; (4) lack of an MSD; and (5) voluntary receipt of URD-HSCT or IST+EPAG as first-line treatment. The exclusion criteria for SAA were (1) congenital bone marrow failure and (2) severe heart, liver or kidney disease.

This was a retrospective, multicenter cohort analysis of patients enrolled from four institutions in China beginning in November 2012 and ending in October 2022. The date of the last follow-up for survivors was April 30, 2023. The study received local review board approval. Written informed consent was obtained from the patients or their caretakers following the Declaration of Helsinki.

URD-HSCT protocol

The conditioning regimen for URD-HSCT included FCA, BUCy and modified PTCy.[2, 3] Specifically, the FCA regimen consisted of fludarabine (Flu, 40 mg/m^2/day from days -7 to -4), cyclophosphamide (Cy, 30 mg/kg/day from days -7 to -4), and rabbit antithymocyte globulin

(rATG, 3.0 mg/kg/day from days -5 to -2). The BuCy regimen consisted of busulfan (Bu, 0.8 mg/kg 4 times daily from days -7 to -6), Cy (50 mg/kg/day from days -5 to -2), and rATG (2.5 mg/kg/day from days -5 to -2). The modified PTCy regimen consisted of Cy (40 mg/kg/day from day -7 to day -6), rATG (2 mg/kg/day from day -5 to day -3), Flu (30 mg/m^2/day from day -5 to day -2) and TBI (4 Gy, day -1) (PTCy-TBI) or, alternatively, Cy (40 mg/kg/day from day -7 to day -6), ATG (2 mg/kg/day from day -5 to day -3), Flu (30 mg/m^2/day from day -5 to day -2) and Bu (0.8 mg/kg 4 times daily from days -4 to -3) (PTCy-Bu).[3]

The patients were separated into matched (HLA 10/10, MUD) and mismatched (HLA 9/10, MMUD) groups by HLA matching at high-level resolution (HLA-A, HLA-B, HLA-C, HLA-DRB1 and HLA-DQB1). Mobilized peripheral blood stem cells (PBSCs) were the only stem cell source, which were infused into the recipient on the day of their collection. As described previously,[2, 4, 5] GVHD prophylaxis included CSA, mycophenolate mofetil (MMF) and methotrexate for patients treated with the FCA and BUCy regimens. The patients treated with the modified PTCy regimen received Cy (40 mg/kg/day on days +3 and +4), MMF and tacrolimus as GVHD prophylaxis.[3] Acute GVHD (aGVHD) and chronic GVHD (cGVHD) were diagnosed and graded according to published criteria.[6, 7]

IST+EPAG protocol

In the IST+EPAG group, the patients received rATG 3-4 mg/kg/day from days 1 to 5, and CSA was administered orally from day 1 of rATG at two separate doses starting at 3 to 5 mg/kg/day; the dose was adjusted to maintain the trough concentration at 150-250 ng/ml, followed by a slow taper until discontinuation (total course of at least two years).[8] EPAG was initiated at a

dose of 50 mg/day on day 1 and was increased by 25 mg every 2 weeks until a maximum of 150 mg or a dose resulting in a hematological response with acceptable tolerability was reached. [8]

Definitions

Myeloid and platelet engraftment were defined as occurring on the first day of an absolute neutrophil count ≥ 0.5 × 10^9/L for three consecutive days and the first day of a platelet count ≥ 20 × 10^9/L for seven consecutive days without transfusion support, respectively. Primary GF (PGF) was defined as ≤ 5% donor chimerism in the bone marrow (BM) or peripheral blood (PB) until day +28 posttransplantation. Initial engraftment and the absence of graft function, followed by recurrent pancytopenia, without moderate to severe aGVHD and loss of donor chimerism, were considered to indicate secondary GF (SGF).[2]

A complete response (CR) was defined as an absolute neutrophil count (ANC) > 1.5 × 10^9/L, hemoglobin > 100 g/L, and platelet count > 100 × 10^9/L (all three criteria had to be met). For SAA and VSAA patients, a partial response (PR) was defined as no longer meeting the criteria for SAA/VSAA and transfusion independence, and no response (NR) was defined as still meeting the criteria for SAA.[1] For NSAA, a PR was defined as transfusion independence or doubling or normalization of at least one cell line, and NR was defined as blood count worsening or not meeting the criteria above.[1] The overall response (OR) rate included the CR and PR rates.

Overall survival (OS) was defined as the time from transplantation or IST+EPAG to death or the last follow-up. Failure-free survival (FFS) was defined as survival with a treatment response.

Death, PGF, SGF, relapse and secondary malignancy were considered treatment failures for HSCT. GVHD-free, failure-free survival (GFFS) was defined as survival without grade III-IV aGVHD, cGVHD requiring systemic treatment, or treatment failure as described above. Death, NR by 6 months and beyond, relapse, clonal evolution and disease progression requiring intervention, whichever occurred first, were considered indicators of treatment failure for IST+EPAG. CROS was defined as survival with a CR status at the last follow-up without treatment failure in the IST+EPAG group.

Evaluation of HRQoL

Patients who survived more than two years were contacted by phone by the investigators as described previously.[9, 10] A survey package including a consent form, a set of questionnaires concerning HRQoL, and a self-addressed stamped envelope was mailed to individuals willing to participate. Each respondent was requested to sign the written informed consent form, complete the HRQoL questionnaires, and return both at their earliest convenience.

The Medical Outcomes Study 36-Item Short-Form Health Survey (SF-36) includes the following eight subscales: physical functioning, role-physical functioning, bodily pain, general health, vitality, social functioning, role-emotional functioning, and mental health. The standard score was transformed from raw scores. Two summary measures, the physical component summary (PCS) and mental component summary (MCS), were aggregated from the eight subscales. [11] High scores indicate high functional levels.

Statistical analysis

The sample size of this retrospective cohort study was estimated according to FFS. With a 2-sided α error of 0.05, at least 164 patients (82 in URD-HSCT and 82 in IST) were necessary for a statistical power of 80% to detect a 20% improvement in FFS in the group receiving upfront URD-HSCT (FFS, 80%) compared with those who received IST (FFS, 60%). Continuous variables are summarized by the median and range. Categorical data are expressed as numbers and percentages. Continuous and categorical variables were compared between the HSCT and IST+EPAG groups by Student's t test (or Mann–Whitney *U* test) and the chi-square test (or Fisher's exact test), respectively. To minimize confounding factors between two groups, propensity score matching (PSM) was applied using 1:1 nearest-neighbor matching with a caliper of 0.2 based on 2 variables (patients age treatment and period of treatment).[12] The cumulative incidence (CuI) of engraftment, aGVHD, cGVHD and GF was estimated using competing risk methods. The probabilities of survival outcomes were calculated according to the Kaplan–Meier method and compared using the log-rank test. Univariate and multivariate analyses were performed using the Cox proportional hazard regression model. A multiple linear regression model was used to analyze HRQoL. The data were analyzed using R version 4.0.1 (http://www.r-project.org).

References

1. Killick SB, Bown N, Cavenagh J, Dokal I, Foukaneli T, Hill A, et al. Guidelines for the diagnosis and management of adult aplastic anaemia. Br J Haematol. 2016;172(2):187-207.
2. Zhang YP, Wu LL, Mo WJ, Zhou M, Li YM, Chen XW, et al. Comparable Outcomes of First-Line Hematopoietic Stem Cell Transplantation from Unrelated and Matched Sibling Donors in Adult Patients with Aplastic Anemia: A Retrospective Single-Center Study. Biol Blood Marrow Transplant. 2019;25(8):1567-75.
3. Wu L, Zhou M, Li Y, Chen X, Mo W, Wang C, et al. Prospective Study of a Modified Post-Transplantation Cyclophosphamide Regimen for Severe Aplastic Anemia Patients with HLA-Haploidentical Transplantation. Transplantation and Cellular Therapy, Official Publication of the American Society for Transplantation and Cellular Therapy. 2023;29(7):463.e1-.e7.
4. Xu LP, Jin S, Wang SQ, Xia LH, Bai H, Gao SJ, et al. Upfront haploidentical transplant for acquired severe aplastic anemia: registry-based comparison with matched related transplant. J Hematol Oncol. 2017;10(1):25.
5. Xu LP, Wang SQ, Wu DP, Wang JM, Gao SJ, Jiang M, et al. Haplo-identical transplantation for acquired severe aplastic anaemia in a multicentre prospective study. Br J Haematol. 2016;175(2):265-74.
6. Przepiorka D, Weisdorf D, Martin P, Klingemann HG, Beatty P, Hows J, et al. 1994 Consensus Conference on Acute GVHD Grading. Bone Marrow Transplant. 1995;15(6):825-8.
7. Filipovich AH, Weisdorf D, Pavletic S, Socie G, Wingard JR, Lee SJ, et al. National Institutes of Health consensus development project on criteria for clinical trials in chronic graft-versus-host disease: I. Diagnosis and staging working group report. Biol Blood Marrow Transplant. 2005;11(12):945-56.
8. Liu L, Lei M, Fu R, Han B, Zhao X, Liu R, et al. Matched related transplantation versus immunosuppressive therapy plus eltrombopag for first-line treatment of severe aplastic anemia: a multicenter, prospective study. J Hematol Oncol. 2022;15(1):105.
9. Liu L, Zhang Y, Jiao W, Zhou H, Wang Q, Jin S, et al. Comparison of efficacy and health-related quality of life of first-line haploidentical hematopoietic stem cell transplantation with unrelated cord blood infusion and first-line immunosuppressive therapy for acquired severe aplastic anemia. Leukemia. 2020;34(12):3359-69.
10. Lei M, Li X, Zhang Y, Qu Q, Jiao W, Zhou H, et al. Comparable Outcomes and Health-Related Quality of Life for Severe Aplastic Anemia: Haploidentical Combined With a Single Cord Blood Unit vs Matched Related Transplants. Front Oncol. 2021;11:714033.
11. Li L, Wang HM, Shen Y. Chinese SF-36 Health Survey: translation, cultural adaptation, validation, and normalisation. Journal of epidemiology and community health. 2003;57(4):259-63.
12. D'Agostino RB, Jr. Propensity score methods for bias reduction in the comparison of a treatment to a non-randomized control group. Statistics in medicine. 1998;17(19):2265-81.

Supplementary Figure legends

Supplementary Figure 1. Transplantation outcomes in SAA patients who underwent URD-HSCT. (A) CuI of neutrophil engraftment. (B) CuI of platelet engraftment. (C) CuI of grade II-III aGVHD. (D) CuI of cGVHD. (E) CuI of grade II-III aGVHD after MUD- and MMUD-HSCT. (F) CuI of cGVHD after MUD- and MMUD-HSCT.

Supplementary Figure 2. Survival outcomes in SAA patients who underwent URD-HSCT. (A) OS in MUD and MMUD groups. (B) GFFS in MUD and MMUD groups.

Supplementary Figure 3. OS in SAA patients who underwent URD-HSCT and IST+EPAG before and after PSM. (A-F) OS in overall and subgroup before PSM. (G-L) OS in overall and subgroup after PSM.

Supplementary Figure 4. Survival outcomes in AA patients who underwent URD-HSCT and IST+EPAG after PSM. (A-F) FFS in overall group and subgroup after PSM. (G-L) GFFS/CROS in overall group and subgroup after PSM.

Supplementary Figure 5. HRQoL in SAA patients who underwent URD-HSCT and IST+EPAG after PSM. PCS, physical component summary; PF, physical functioning; RF, role-physical functioning; BP, bodily pain; GH, general health; MCS, mental component summary; SF, social functioning; RF, role-emotional functioning; MH, mental health.

Supplementary Figure 1

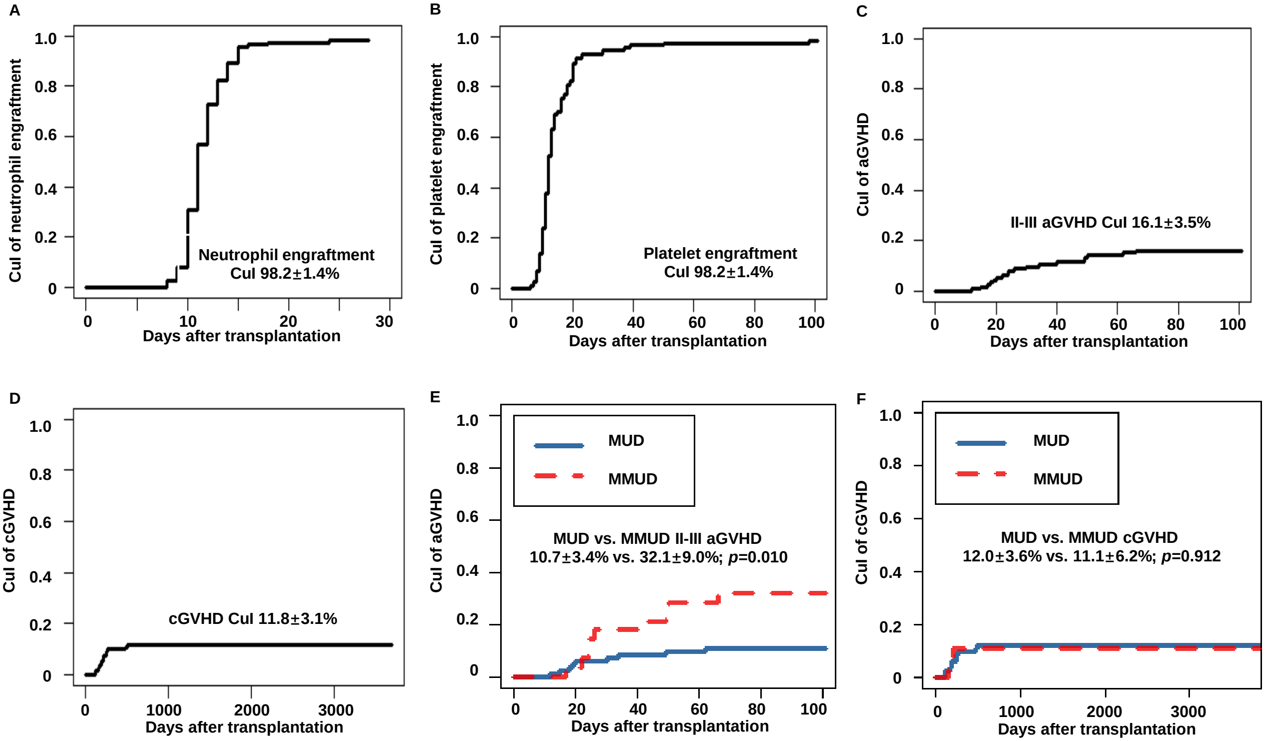

Supplementary Figure 2

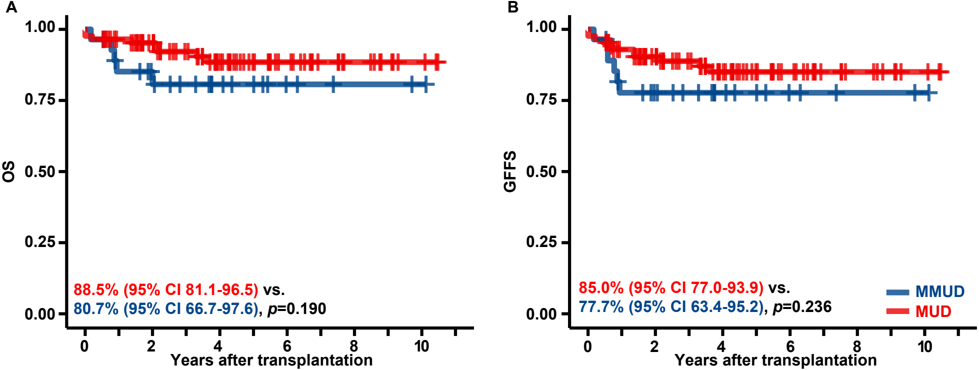

Supplementary Figure 3

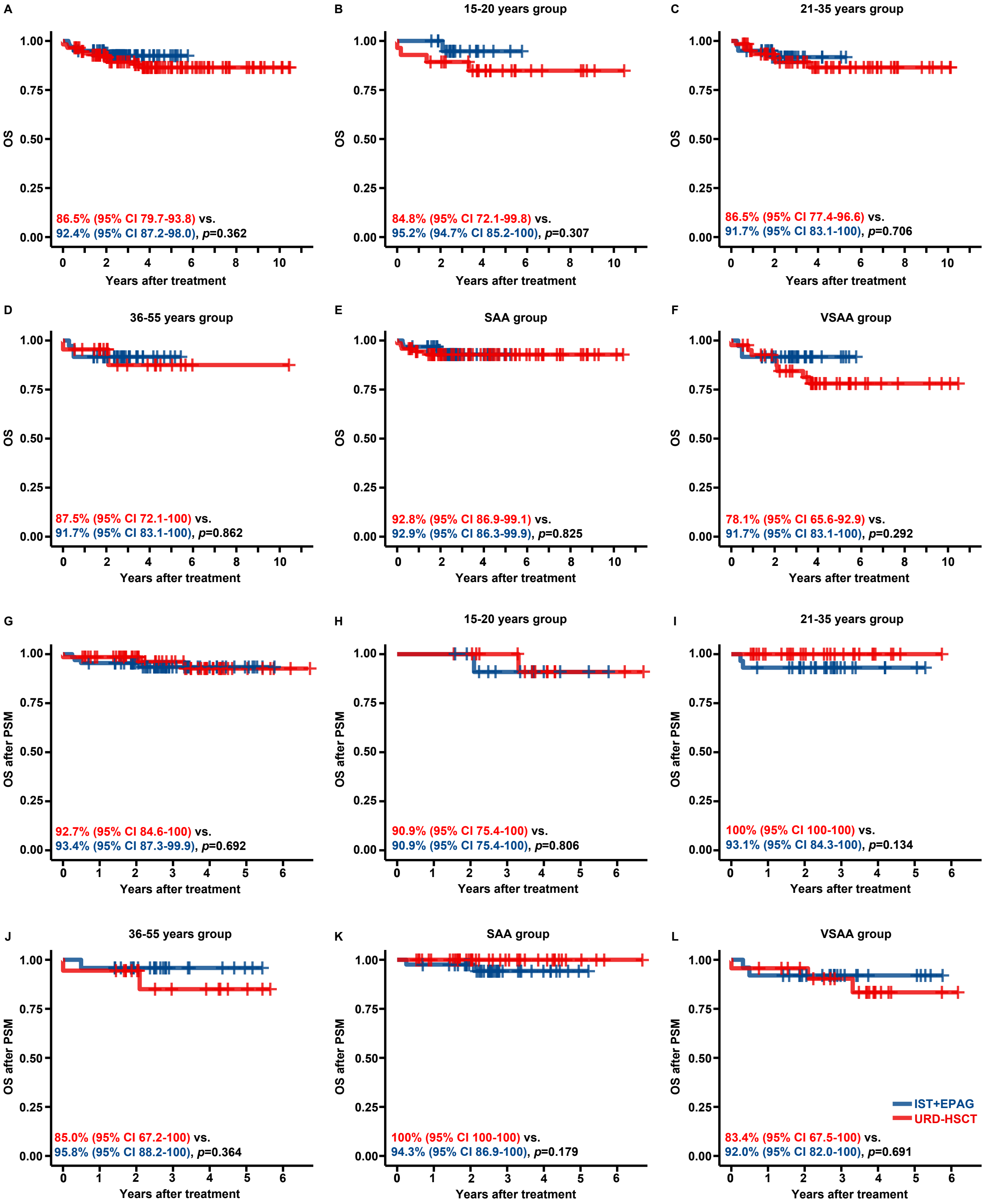

Supplementary Figure 4

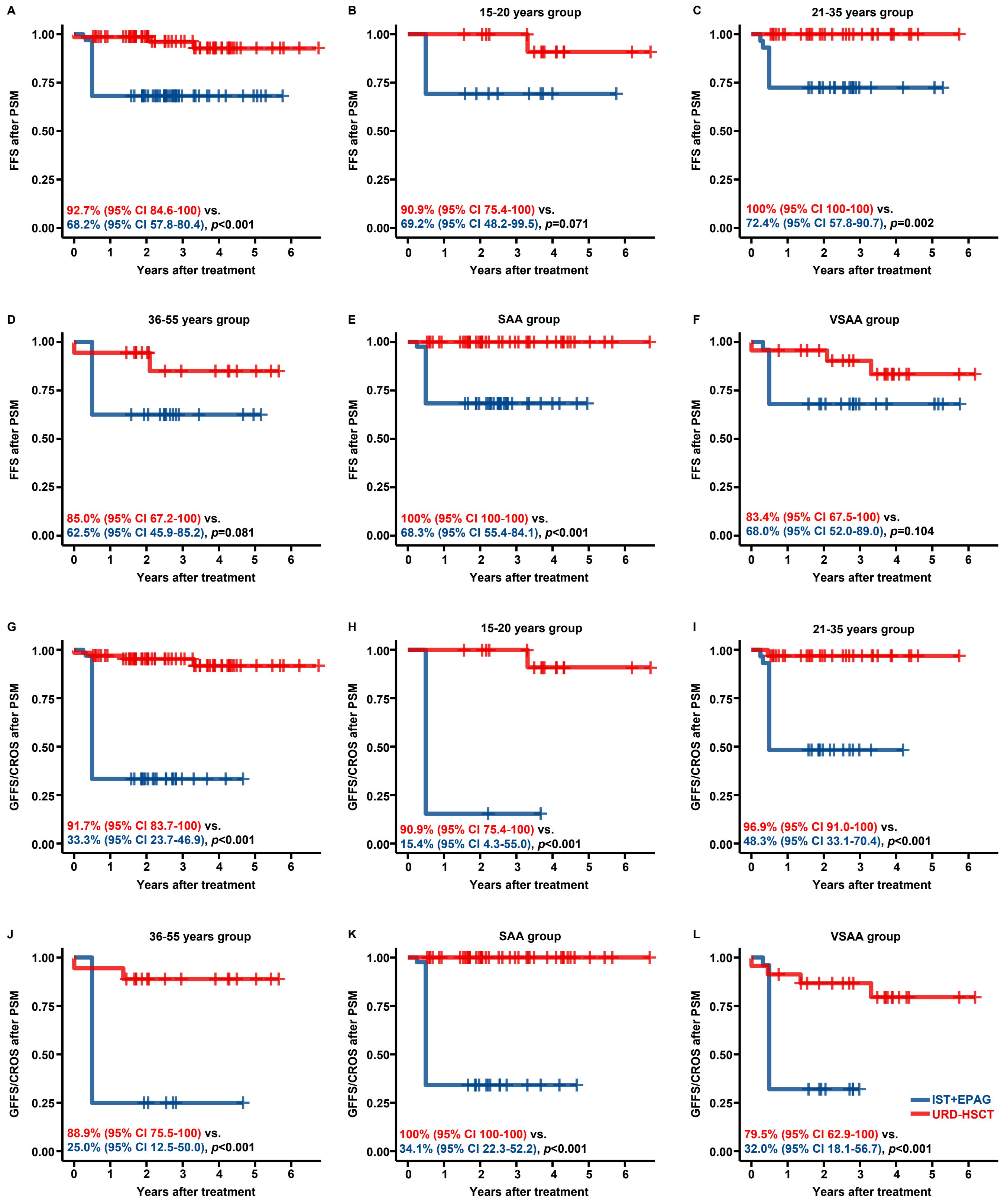

Supplementary Figure 5

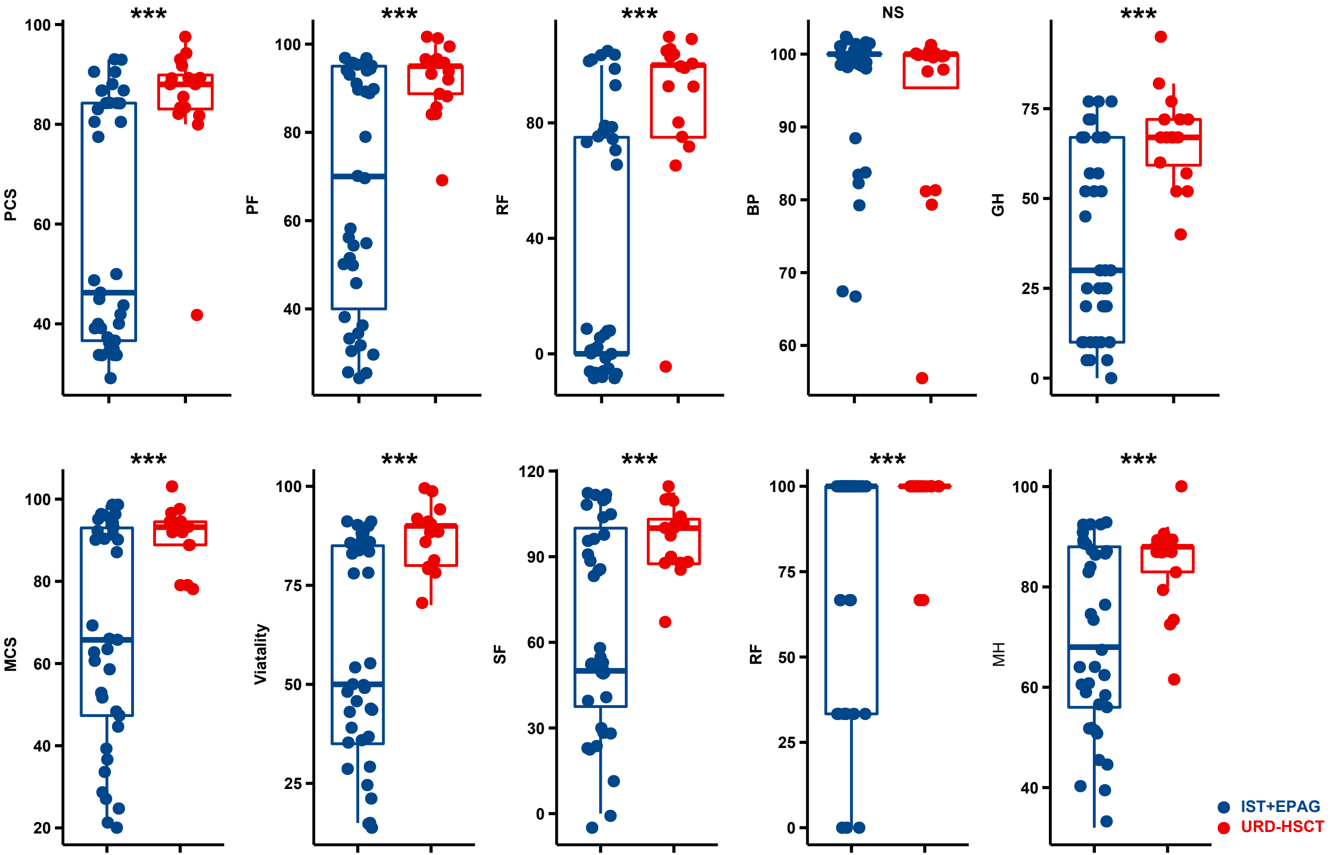

Supplementary Table 1. Subgroup analysis of associations between treatment choice and survival outcomes

Characteristics	IST+EPAG	URD-HSCT# HR (95% CI)	*P* #	URD-HSCT* HR (95% CI)	*P* *
OS					
Age, y					
15-20	1	2.290 (0.330-27.068)	0.330	0.707 (0.044-11.348)	0.827
21-35	1	1.301 (0.331-5.117)	0.707	-	-
36-55	1	1.165 (0.194-6.989)	0.867	2.891 (0.261-31.932)	0.386
Diagnosis					
SAA	1	1.160 (0.311-4.321)	0.825	-	-
VSAA	1	2.013 (0.530-7.639)	0.304	1.437 (0.238-8.691)	0.693
FFS					
Age, y					
15-20	1	0.724 (0.178-2.945)	0.651	0.161 (0.018-1.455)	0.104
21-35	1	0.281 (0.109-0.729)	0.009	-	-
36-55	1	0.168 (0.039-0.732)	0.018	0.262 (0.056-1.214)	0.868
Diagnosis					
SAA	1	0.245 (0.092-0.653)	0.005	-	-
VSAA	1	0.219 (0.081-0.0.591)	0.003	0.331 (0.087-1.258)	0.105
GFFS/CROS					
Age, y					
15-20	1	0.143 (0.050-0.409)	<0.001	0.085 (0.019-0.372)	0.001
21-35	1	0.145 (0.068-0.308)	<0.001	0.113 (0.027-0.475)	0.003
36-55	1	0.059 (0.014-0.252)	<0.001	0.178 (0.063-0.504)	0.001
Diagnosis					
SAA	1	0.073 (0.031-0.173)	<0.001	-	-
VSAA	1	0.173 (0.082-0.363)	<0.001	0.237 (0.099-0.570)	0.001

#, before PSM; , after PSM.

Supplementary Table 2. Univariate and multivariable analyses of risk factors for OS, FFS and GFFS/CROS

Risk Factor	Before PSM				After PSM			
	Univariate analysis		Multivariable analysis		Univariate analysis		Multivariable analysis	
	Hazard Ratio (95% CI)	*P*	Hazard Ratio (95% CI)	*P*	Hazard Ratio (95% CI)	*P*	Hazard Ratio (95% CI)	*P*
OS								
Age, y								
15-20	1		1		1		1	
21-35	1.076 (0.368-3.153)	0.893	0.946 (0.319-2.805)	0.921	0.609 (0.084-4.383)	0.622	0.648 (0.089-4.721)	0.668
36-55	0.986 (0.285-3.412)	0.982	0.905 (0.247-3.317)	0.881	1.284 (0.213-7.751)	0.785	1.040 (0.158-6.381)	0.968
Gender								
female	1		1		1		1	
male	0.407 (0.162-1.019)	0.055	0.438 (0.170-1.132)	0.088	0.144 (0.017-1.195)	0.073	0.140 (0.015-1.299)	0.084
Diagnosis								
NSAA+SAA	1		1		1		1	
VSAA	1.996 (0.826-4.821)	0.125	1.707 (0.682-4.278)	0.254	4.061 (0.786-20.986)	0.094	4.312 (0.790-23.535)	0.091
Course, m								
<3	1		1		1		1	
⩾3	0.909 (0.376-2.199)	0.833	0.777 (0.261-2.314)	0.650	0.733 (0.164-3.284)	0.685	1.757 (0.108-28.640)	0.692
Period of treatment								
11/2012-12/2017	1		1		1		1	
1/2018-10/2022	0.377 (0.1155-0.921	0.032	0.443 (0.159-1.233)	0.119	0.511 (0.060-4.327)	0.538	1.155 (0.130-10.266)	0.897
Treatment								
IST+EPAG	1		1		1		1	
URD-HSCT	1.533 (0.608-3.866)	0.366	1.247 (0.346-4.493)	0.736	0.739 (0.165-3.320)	0.694	0.530 (0.034-8.354)	0.652
FFS								
Age, y								
15-20	1		1		1			
21-35	1.203 (0.527-2.749)	0.661	1.416 (0.614-3.263)	0.414	0.816 (0.266-2.502)	0.722	0.928 (0.293-2.940)	0.568
36-55	2.277 (0.989-5.245)	0.053	1.940 (0.818-4.604)	0.133	1.693 (0.587-4.884)	0.330	1.277 (0.427-2.824)	0.662
Gender								
female	1		1		1		1	
male	0.720 (0.401-1.291)	0.270	0.855 (0.458-1.594)	0.621	0.506 (0.221-1.157)	0.107	0.487 (0.187-1.270)	0.141
Diagnosis								

NSAA+SAA	1		1		1		1	
VSAA	1.644 (0.916-2.950)	0.096	1.535 (0.841-2.800)	0.163	1.483 (0.664-3.314)	0.337	1.376 (0.595-3.182)	0.456
Course, m								
<3	1		1		1		1	
⩾3	0.416 (0.218-0.794)	0.008	1.048 (0.453-2.425)	0.913	0.302 (0.120-0.763)	0.011	1.814 (0.518-6.354)	0.352
Period of treatment								
11/2012-12/2017	1		1		1		1	
1/2018-10/2022	1.049 (0.516-2.131)	0.895	0.420 (0.174-1.013)	0.053	0.746 (0.175-3.189)	0.693	0.807 (0.167-3.893)	0.790
Treatment								
IST+EPAG	1		**1**		1		1	
URD-HSCT	0.285 (0.149-0.547)	<0.001	**0.204 (0.077-0.542)**	**0.001**	0.118 (0.035-0.397)	<0.001	**0.077 (0.016-0.369)**	**0.001**
GFFS/CROS								
Age, y								
15-20	1		1		1		1	
21-35	0.779 (0.453-1.338)	0.365	0.883 (0.513-1.521)	0.655	0.622 (0.294-1.317)	0.215	0.495 (0.229-1.068)	0.073
36-55	1.494 (0.858-2.602)	0.156	1.161 (0.648-2.080)	0.616	1.246 (0.609-2.551)	0.547	0.922 (0.435-1.953)	0.832
Gender								
female	1		1		1		1	
male	0.896 (0.588-1.366)	0.610	1.003 (0.639-1.574)	0.991	0.974 (0.553-1.716)	0.927	1.153 (0.602-2.209)	0.667
Diagnosis								
NSAA+SAA	1		1		1		1	
VSAA	1.292 (0.845-1.976)	0.238	1.207 (0.774-1.881)	0.407	1.457 (0.824-2.578)	0.196	1.284 (0.707-2..333)	0.412
Course, m								
<3	1		1		1		1	
⩾3	0.271 (0.166-0.445)	<0.001	1.167 (0.621-2.193)	0.631	0.230 (0.117-0.452)	<0.001	1.425 (0.622-3.262)	0.403
Period of treatment								
11/2012-12/2017	1		1		1		1	
1/2018-10/2022	1.722 (0.968-3.060)	0.064	**0.408 (0.199-0.837)**	**0.015**	1.008 (0.313-3.251)	0.989	0.751 (0.214-2.639)	0.656
Treatment								
IST+EPAG	1		**1**		1		1	
URD-HSCT	0.115 (0.067-0.199)	<0.001	**0.073 (0.033-0.163)**	**<0.001**	0.054 (0.019-0.153)	<0.001	**0.039 (0.012-0.132)**	**<0.001**

Supplementary Table 3. SF-36 scores of survivors by treatment

	Before PSM			After PSM		
	**IST+EPAG (n=56)**	**URD-HSCT (n=33)**	***P***	**IST+EPAG (n=37)**	**URD-HSCT (n=16)**	***P***
**Phsical, mean (SD)**						
Physical component summary	53.7 (23.5)	82.3 (15.1)	<0.001	59.1 (24.2)	84.9 (12.5)	<0.001
Physical functioning	59.4 (27.0)	90.0 (13.9)	<0.001	66.1 (27.1)	91.6 (7.69)	<0.001
Role-physical functioning	29.0 (41.5)	81.1 (26.5)	<0.001	37.2 (43.9)	87.5 (25.8)	<0.001
Bodily pain	94.8 (9.49)	95.3 (12.8)	0.840	96.0 (8.90)	93.8 (12.3)	0.536
General health	31.7 (24.2)	62.7 (14.8)	<0.001	37.0 (25.8)	66.6 (12.9)	<0.001
**Psychological, mean (SD)**						
Mental component summary	61.5 (26.4)	91.8 (9.75)	<0.001	66.9 (26.7)	91.2 (7.01)	<0.001
Vitality	52.9 (25.6)	88.9 (10.7)	<0.001	58.5 (26.6)	87.5 (7.96)	<0.001
Social functioning	56.7 (35.5)	92.8 (22.3)	<0.001	63.9 (35.9)	96.9 (13.3)	<0.001
Role-emotional functioning	70.8 (37.1)	98.0 (8.08)	<0.001	75.7 (34.8)	95.8 (11.4)	0.003
Mental-health	65.5 (18.1)	87.4 (9.60)	<0.001	69.7 (18.6)	84.5 (9.45)	<0.001

Supplementary Table 4. Multivariate analysis of favorable factors for HRQoL

Outcome in URD-HSCT	Before PSM		After PSM	
	Regression coefficient	*P*	Regression coefficient	*P*
Physical component summary	36.470	<0.001	35.929	<0.001
Physical functioning	37.688	<0.001	37.436	<0.001
Role-physical functioning	68.093	<0.001	68.349	<0.001
General health	40.244	<0.001	39.439	<0.001
Mental component summary	34.701	<0.001	35.357	<0.001
Vitality	39.172	<0.001	38.228	<0.001
Social functioning	47.196	<0.001	47.010	<0.001
Role-emotional functioning	29.909	0.002	34.297	0.002
Mental-health	22.529	<0.001	21.892	0.001

Supplementary Table 5. SF-36 scores of survivors by hematologic response in the two groups

	Before PSM			After PSM		
	CR (n=44)	PR (n=31)	*P*	CR (n=26)	PR (n=20)	*P*
**Phsical, mean (SD)**						
Physical component summary	83.9 (11.6)	50.1 (20.7)	<0.001	86.2 (4.83)	52.5 (22.9)	<0.001
Physical functioning	91.4 (11.7)	58.9 (22.4)	<0.001	93.1 (4.26)	63.2 (22.5)	<0.001
Role-physical functioning	84.1 (20.2)	19.4 (36.9)	<0.001	88.5 (12.7)	23.8 (42.5)	<0.001
Bodily pain	97.5 (9.43)	92.4 (12.1)	0.057	97.9 (6.03)	91.0 (13.6)	0.048
General health	62.5 (13.0)	29.6 (23.1)	<0.001	65.2 (10.7)	31.8 (25.7)	<0.001
**Psychological, mean (SD)**						
Mental component summary	92.5 (8.42)	60.3 (22.0)	<0.001	92.3 (5.17)	62.2 (24.3)	<0.001
Vitality	88.3 (9.02)	53.2 (20.2)	<0.001	87.3 (5.70)	54.8 (22.5)	<0.001
Social functioning	95.7 (20.0)	55.2 (27.2)	<0.001	98.6 (10.2)	58.8 (31.7)	<0.001
Role-emotional functioning	98.5 (7.02)	67.7 (37.0)	<0.001	97.4 (9.06)	68.3 (36.6)	0.002
Mental-health	87.5 (8.51)	65.0 (15.1)	<0.001	86.0 (7.94)	66.8 (16.6)	<0.001
